# Protocol for an implementation study of group metacognitive therapy for anxiety and depression in NHS cardiac rehabilitation services in England (PATHWAY-Beacons)

**DOI:** 10.3389/frhs.2024.1296596

**Published:** 2024-10-17

**Authors:** Adrian Wells, David Reeves, Andrew Belcher, Paul Wilson, Patrick Doherty, Lora Capobianco

**Affiliations:** ^1^ Division of Psychology and Mental Health, School of Psychological Sciences, Faculty of Biology, Medicine and Health, School of Health Sciences, The University of Manchester, Manchester, United Kingdom; ^2^ Research and Innovation, Greater Manchester Mental Health NHS Foundation Trust, National Health Service Foundation, Manchester, United Kingdom; ^3^ NIHR School for Primary Care Research, Williamson Building, Manchester Academic Health Science Centre, The University of Manchester, Manchester, United Kingdom; ^4^ Jean McFarlane Building, Faculty of Biology Medicine and Health, Centre for Biostatistics, School of Health Sciences, The University of Manchester, Manchester, United Kingdom; ^5^ Division of Population Health, Health Services Research and Primary Care, School of Health Sciences, University of Manchester, Manchester, United Kingdom; ^6^ Department of Health Sciences, University of York, York, United Kingdom

**Keywords:** cardiac rehabilitation, metacognitive therapy, mental health, implementation, anxiety

## Abstract

**Background:**

Cardiac rehabilitation (CR) services aim to improve heart disease patients’ health and quality of life and reduce the risk of further cardiac events. Depression and anxiety are common among CR patients but psychological treatments have usually had small effects. In contrast, the recent NIHR-funded PATHWAY trial found that group Metacognitive Therapy (MCT) was associated with improvements in anxiety and depression when added to CR and was more effective than usual CR alone. The next stage is to test implementation of MCT within the National Health Service through the creation of a network of CR beacon sites. The study will test the quality of data capture following addition of a new MCT data-field to the national audit of cardiac rehabilitation (NACR), examine level of adoption at sites, examine mental health outcomes benchmarked against usual CR and the PATHWAY data, examine the enablers and barriers to implementation and the expected resource requirements. The study has been registered: NCT05956912 (13th July, 2023).

**Methods:**

Beacon sites will be recruited as preliminary adopters of group MCT from NHS CR services in England. A national invitation for expressions of interest from CR services will be issued and those meeting eligibility criteria will be considered for inclusion. Two staff at each site will receive training in MCT, and mixed-methods will be used to address questions concerning the quality of patient data recorded, level of adoption at sites, the characteristics of patients attending MCT, the impact of adding MCT to CR on mental health outcomes, and patient, healthcare staff and commissioner views of barriers/enablers to implementation. Exploration of implementation will be informed by Normalisation Process Theory.

**Discussion:**

The study will support development of an NHS roll-out strategy, assess the mental health outcomes associated with MCT, examine treatment fidelity in real-world settings, and evaluate revised data collection structures that can be used to assess the impact of national-level implementation.

**Trial Registration:**

NCT05956912; 13^th^ July 2023.

## Background

Cardiovascular disease (CVD) is the most common non-communicable disease and the largest contributor to morbidity and mortality worldwide (1, 2). Cardiac rehabilitation (CR) programmes aim to facilitate recovery after a heart event, promote healthy behaviours, improve lifestyle risk factors, reduce the risk of further related problems, and improve patients' emotional well-being (e.g., anxiety, depression) and health-related quality of life.

The psychological burden of CVD is well known (2–5). Anxiety and depression are common, affecting approximately 30% of CVD patients (6), and are associated with decreased treatment adherence, poor lifestyle factors (e.g., smoking), poorer quality of life, higher healthcare costs and readmission, increased risk of mortality, and poorer long-term psychological adjustment (2–13). The European Association of Preventive Cardiology has emphasised that symptoms of anxiety and depression in heart disease patients affect the success of CR programmes (14). A recent UK-based study of depression in patients attending CR in the pre and post Covid era reaffirmed the need to prioritise psychological well-being (15). As such, it is imperative to identify and treat symptoms of anxiety and depression effectively to ensure that CR programmes have better clinical outcomes, improve the quality of life of heart disease patients and reduce health service costs.

The effect of interventions to treat anxiety and depression (e.g., pharmacotherapy, psychological therapies) in CVD have produced mixed and often small effects (16–21). The most recent Cochrane review (20) and meta-analysis included 35 randomised controlled trials of 10,703 people with CVD treated for anxiety and depression compared to usual care. Psychological treatments included relaxation techniques, emotional support or client-led discussion, cognitive challenging or cognitive restructuring techniques, and other psychological approaches (i.e., stress management/psychotherapy). There was evidence of small reductions in anxiety (pooled SMD −0.24, 95% CI: −0.09 to −0.38) and depression (pooled SMD −0.27, 95% CI: −0.15 to −0.39) at a median follow-up of 12 months in favour of the intervention. However, insufficient evidence quality means considerable uncertainty about the observed effects exists.

Recently, the National Institute for Health and Care Research (NIHR) funded the PATHWAY Trial (RP-PG-1211-20011) to evaluate for the first-time group metacognitive therapy (MCT) for anxiety and depression in cardiac rehabilitation. MCT is based on a psychological model where anxiety and depression are maintained by common factors, including negative repetitive thinking (i.e., worry, rumination) and threat monitoring. Group MCT is a manualised treatment approach that may be particularly suited to addressing these factors in CR patients. Unlike other therapies, MCT does not require an in-depth analysis and challenging of the content of patients' worries, that in the CR context are often realistic (22, 23). Instead, it focuses on enabling patients to effectively regulate worry, rumination, and other unhelpful behaviours that maintain anxiety and depression. A pilot feasibility trial to assess the acceptability and feasibility of adding group MCT to CR (24) found that adding group MCT to CR did not reduce attendance at routine CR, with 78% of patients attending group MCT. A full-scale randomised controlled trial that followed demonstrated that adding MCT to CR was associated with improved mental health outcomes beyond those obtained in usual care, with lower anxiety and depression (total HADS) at 4-month follow-up [SMD = 0.52 (0.29–0.75), *p* < 0.011], which was maintained at twelve-months [SMD = 0.33 (0.10–0.57), *p* = 0.01]. An incidental finding was that MCT also appeared to reduce the risk of psychological deterioration (25).

In view of these results and the identified need to improve psychological outcomes in cardiac patients, the next step involves evaluating implementation and possible roll-out of group MCT across CR services in the UK National Health Service (NHS). There are multiple challenges to developing and successfully implementing new treatments in practice. Challenges include training a workforce to effectively deliver complex interventions, facilitating patient knowledge and uptake, and ensuring treatment quality, engagement, and participation of stakeholders.

The implementation methodology used in this study is informed by Normalisation Process Theory [NPT; (26)]. NPT (27–31) concerns three core factors and the processes underpinning them: Implementation (introducing an intervention into action), embedding (routine incorporation of an intervention into the everyday work of individuals and groups), and integration (reproduction and sustainability of the intervention in organisations and institutions). NPT focuses on the work that individuals and groups do to enable an intervention to become normalised. Four main components to NPT provide a framework for understanding the factors that facilitate and inhibit implementation: (1) coherence building (sense making), understanding what makes an intervention meaningful; (2) cognitive participation (or engagement), which forms commitment around an intervention (e.g., How do individuals collaborate to establish engagement networks and communities of practice around the intervention?); (3) collective action, understanding how individuals work together to enact the intervention; and (4) reflexive monitoring, which concerns how interventions and their components are appraised.

The initial objectives are to establish and develop a network of NHS beacon sites offering group MCT and to revise and pilot national data monitoring to capture group MCT outcomes.

To facilitate and evaluate implementation quantitative and qualitative methods will be used to address specific questions:

1) What is the quality of data capture and monitoring for group MCT?
2) What is the level of adoption of group MCT at each Beacon site and across all sites combined?
3) How do patients attending MCT + CR differ from those participating in CR only in terms of sociodemographic and health characteristics?
4) What impact does adding group MCT to CR have on mental health and quality of life outcomes benchmarked against CR alone and against the outcomes for MCT plus CR in the PATHWAY trial?
5) What are CR staff's views of group MCT training and delivery, and what are the facilitators and barriers?
6) What is the acceptability and feasibility of roll-out at the commissioner level, and what are the facilitators or barriers?
7) What are the expected and unexpected consequences of implementing group MCT, for example, on existing CR services, staffing needs, administration, and resource requirements?

## Methods

### Study overview

The study will establish “beacon sites” that will provide a network for wider-scale evaluation of roll-out in the NHS. Two therapists will be trained to deliver group MCT alongside CR at each of the participating sites.

A new data-field in National Audit of Cardiac Rehabilitation (NACR) database will be created to aid with data collection on the adoption of group MCT in CR. Routine Hospital Anxiety and Depression Scale (HADS) (32) data will be collected via the NACR database, which routinely contains HADS on patients pre- and post-CR.

Semi-structured interviews will be conducted with CR staff trained in MCT and CR stakeholders to assess the implementation of group MCT within CR services.

Our success criteria are:

1) Establishment of a minimum of three new beacon sites willing and able to support a large-scale roll-out study.
2) Addition of an MCT field in the National Audit of Cardiac Rehabilitation (NACR) audit system and preliminary data on the quality of capturing group MCT related outcomes.
3) Uptake of group MCT within services at each site will be based on delivering at least two six-session group MCT courses with 3–10 patients per group, with at least 60% attending at least four sessions.
4) Identification of barriers and facilitators to implementation in the NHS in the areas of: - how is the intervention understood - how can it be translated into practice - how will it fit into services - how will it be sustained.

### Site recruitment

At least three new Beacon sites, not previously involved in the PATHWAY trial, will be recruited as preliminary adopters of group MCT from NHS CR services in England. Previous sites involved in the PATHWAY trial will also be invited to continue their involvement with MCT-PATHWAY. In addition, we will recruit new CR services. UK CR services will be invited to express an interest in taking part in the adoption study, and express an interest by completing an expression of interest form. CR services who express an interest will be reviewed to assess if they meet the eligibility criteria, and those meeting the eligibility criteria will be invited to participate. The sites will be chosen based on the level of CR referrals and the flexibility of CR staff to engage in at least three days of training to deliver the intervention.

To be eligible, the sites must be:

1) NACR registered and achieved minimum of current amber status on the National Certification Programme for CR (NCP_CR) which is jointly run by the NACR and the British Association for Cardiovascular Prevention and Rehabilitation (BACPR)
2) Agree to deliver group MCT to a minimum of two groups (including the pilot therapy group as part of training).
3) Release two CR staff for three days of online training, deliver two groups (six sessions × 90 min per group) and take half a day to participate in qualitative interviews.
4) CR services must not be participating in any other service evaluations.

### Participants

Beacons sites must identify CR staff members to be trained to deliver MCT. Eligible staff must be healthcare professionals working with cardiac rehabilitation services. All CR staff trained in group MCT will be invited to participate in the interviews; before, during and after MCT training. Post-training interviews will provide a reflective approach to implementation and experience in the delivery of MCT.

In addition to interviewing staff trained in MCT, a range of stakeholders (i.e., CR managers, commissioners) will be invited to take part in qualitative interviews. To be eligible to participate in semi-structured interviews, staff must be a healthcare professional working with cardiac rehabilitation or a professional working at the commissioner level. CR stakeholders will be invited to participate in an interview and recruited from CR services, commissioning groups and other relevant CR stakeholder areas.

### Implementation

Designated CR practitioners at each site will attend three days of online training sessions delivered by the study team at Greater Manchester Mental Health NHS Foundation Trust. Training will be led by AW, the developer of MCT. The training programme will include didactic teaching, watching MCT training videos and role plays, studying the intervention manual, and conducting a pilot therapy group. The first two training days will precede the implementation of the pilot training group. The third training day will follow the pilot group to review any challenges and conduct further training. A detailed treatment manual developed in the previously funded NIHR PATHWAY trial will guide CR professionals in delivering group MCT.

### Group MCT patient eligibility

All patients attending CR who meet the NICE recommendations for acute coronary syndrome (NG185) (33) and heart failure (NG106) (34) will be offered group MCT as part of routine CR. Patients will be invited to participate in group MCT during their assessment appointment. CR practitioners were provided guidance from the research team on how to offer group-MCT to interested and eligible CR patients. In addition, interested and eligible participants will be offered an information booklet on group-MCT which was co-created by the study patient and public involvement group. A minimum of three participants will be required to conduct group MCT with a maximum of 10 participants per group. Participants interested in participating in group MCT will be provided with the dates and times of the sessions. Group MCT will run in six-week blocks alongside CR.

### Intervention

Group MCT is a brief group-based psychological treatment to reduce anxiety and depression symptoms in patients attending CR. The intervention is evidence-based and supported by a structured treatment manual. The intervention consists of six sessions of approximately 90 min and will be delivered face-to-face. It presents a series of structured group exercises to explore and modify thinking patterns and behaviours that keep anxiety and depression going. The treatment approach identifies a pattern of excessive thinking in the form of worry, rumination, and focusing on threats that maintain anxiety and depression and interferes with adjustment to stressful situations. Group MCT aims to reduce this pattern by mapping out and modifying underlying psychological factors (metacognition) involved in regulating thinking. For further details on the intervention and its effectiveness, see Wells et al. (22).

### Data collection

We will add a rehabilitation delivery data field to the NACR database to include uptake of group MCT. In addition, routine outcomes from the NACR database will be collected, including anxiety and depression outcome data (i.e., the Hospital Anxiety and Depression scale), quality of life (i.e., Dartmouth Co-op), and clinical and demographic characteristics. Data will be captured from sites on attendance rates. In addition, CR staff will be asked to complete an adherence checklist to monitor adherence to the treatment protocol.

Qualitative data will be collected through semi-structured interviews completed either face-to-face, via Microsoft Teams, or by telephone. Interviews will follow a topic guide and will be audio recorded, anonymised, and transcribed verbatim. The interviews will be conducted with approximately 10 CR staff members trained in group MCT and 10 CR stakeholders (CR nurses, physiotherapists, occupational therapists, managers, and commissioners).

Participants will be prompted about their perspectives across four domains based on NPT, which include: sense-making (how the intervention is understood and compared with existing practices), implementation (how it is translated into practice), embedding (how will it fit routinely within services), and integration (how will it be sustained as part of routine practice).

### Clinical outcome measures

The Hospital Anxiety and depression Scale [HADS (32)] is a 14-item self-report questionnaire that measures symptoms of anxiety (7 items) and depression (7 items). Items are rated using a 4-point (0–3) scale, with higher scores indicating elevated distress. Scores for each subscale range from 0 to 21 and can be categorised as normal (0–7), mild (8–10), moderate (11–14), or severe (15–21). The NACR pays an annual licence fee for HADS so that all NHS based CR programmes can use it free of charge.

The Dartmouth COOP (35) is a self-report measure designed to evaluate the functional abilities of medical patients. It consists of nine items covering various areas such as physical function, daily activities, pain, social activities, social support, emotions, overall health, changes in health, and quality of life. Each chart includes text and illustrations to assist the user in responding. The responses are graded on an ordinal scale of 1–5, with one being the best score.

### Implementation outcomes

Successful adoption of group MCT at each site is set a-priori as the delivery of two, 6-session group MCT courses with three to ten patients per group, with at least 60% attending a minimum of four sessions. Attendance of a minimum of four sessions was selected as this has previously been defined as the minimum treatment dose (36).

A traffic-light criteria will be used to determine successful adoption.

1. Green – achieved all of the following: (a) Delivered two 6-session group MCT courses, with (b) at least three patients per group, and (c) with a combined total of at least 60% attending four or more sessions.
2. Amber - achieved one of the three criteria given above.
3. Red - achieved none of the criteria.

A green rating indicates that a site has fully met the criteria for successful adoption. Amber is considered partial adoption, where amendments to procedures and protocols might lead to successful adoption. At amber sites, specific qualitative work will be undertaken to understand the reasons for not meeting criteria more fully and to help develop strategies to ameliorate this. Red indicates a failure to meet the requirements.

Semi-structured interviews will be conducted with CR staff trained in MCT and CR stakeholders to assess the implementation of group MCT within CR services. In-depth interviews will follow a topic guide to identify the facilitators and barriers to implementation amongst service users, staff, and commissioners, which was developed and guided by the NPT framework. Data from these activities will be used to determine the mechanisms and processes required to support the adoption of training and implementation. In addition, semi-structured interviews will be conducted with CR staff training in and delivering MCT to assess their view on training and delivery.

### Data analysis

#### Quantitative analysis

The Beacon sites and the NACR database will record patient attendance at group MCT. To assess if the modification to the NACR database successfully captures MCT attendance, descriptive statistics will be used to compare the data on patient attendance collected at the site level with that recorded in the NACR dataset.

To assess implementation of MCT at CR services an overall assessment will be made considering the traffic-light ratings of all beacon sites.

The NACR dataset collects a range of demographic, clinical, functional, and physiological measures on each CR patient, at both entry into CR and at discharge. The NACR data at entry will be used to describe the cohort of patients attending MCT and to compare their baseline characteristics to patients on CR only. The NACR data collected at discharge will be used to compare patient outcomes (HADS and Dartmouth Co-op etc.) between the cohorts (MCT + CR vs. CR alone). The analysis will control for a pre-specified set of baseline (entry) confounders: site, HADS, sex, age, number of comorbidities and number of previous different cardiovascular events. Sensitivity analysis will control for additional covariates that differed between the groups (MCT + CR vs. CR alone) above a specified threshold at entry. Further analysis will adjust for missing outcome scores at discharge. Caution will be exercised when interpreting the results as patients have not been randomised to groups hence systematic differences may exist due to unmeasured confounding factors. Therapist adherence to the MCT manual will be assessed using a checklist recording the treatment components implemented in each session, if any components had been missed, and if so, why. The list includes six to eight items for each session, covering the main techniques administered in each session. Session adherence scores will be computed by counting the total elements completed. Descriptive statistics will be used to assess adherence to the group MCT manual across Beacon sites. In addition, change in adherence over time will be compared between sites.

#### Qualitative analysis

Qualitative analysis will use thematic analysis as outlined by Braun and Clarke (37). Initial codes will be genereated inductively, and not driven by a pre-determined framework. Each transcript will be coded based on the study aims. We will subsequently draw on the NPT framework (26) to help consider implications for assimilating the intervention into existing practice. The researcher will lead the analysis, using constant comparison to cycle between data and the developing analytic framework, working with a core analysis team (research assistant/research fellow) who will read all transcripts. The analysis will be tested and developed by discussion regularly with the broader study team.

## Discussion

MCT is a psychological intervention that has the potential to significantly improve psychological outcomes in patients with CVD and offer added choice and value over standard healthcare practices. The mental health benefits would be substantial if MCT could be provided to the 90,000 patients in the United Kingdom commencing CR annually. However, there are significant recognized challenges to implementing and rolling out complex interventions of this kind. We propose a study that will allow us to create the data-monitoring structure and knowledgebase across beacon sites and evaluate data quality and the real-world effects of implementing MCT. The findings will allow us to adapt and modify data capture and monitoring and better understand group MCT training and delivery to facilitate its implementation in cardiac rehabilitation services. The use of quantitative and qualitative methods will generate data to inform and prepare for potential roll-out and evaluation across CR services in the NHS.

Our quantitative work will provide data on the quality of data-capture, the level of adoption at NHS sites, adherence to the treatment protocol and sociodemographic and heath differences between patients who take-up MCT and those who do not. It will assess performance on key outcomes benchmarked against treatment as usual; however, these results must be interpreted with caution: there will be no randomisation hence estimated group differences – even after control for known factors – will be subject to unmeasured confounding. The qualitative stream will develop an account of the influences on training and barriers and facilitators to implementation that has implications for maximising engagement. Use of the NPT framework will facilitate examination of the factors important for embedding MCT within existing services.

## References

[1] World Health Organization (WHO). WHO Global NCD Action Plan 2013–2020 [Internet]. 2013 [cited 2023 12]. Available online at: https://www.who.int/nmh/events/ncd_action_plan/en/

[2] Muka T Imo D Jaspers L Colpani V Chaker L van der Lee SJ The global impact of non-communicable diseases on healthcare spending and national income: a systematic review. Eur J Epidemiol. (2015) 30(4):251–77. 10.1007/s10654-014-9984-2 25595318

[3] Meijer A Conradi HJ Bos EH Thombs BD van Melle JP de Jonge P. Prognostic association of depression following myocardial infarction with mortality and cardiovascular events: a meta-analysis of 25 years of research. Gen Hosp Psychiatry. (2011) 33(3):203–16. 10.1016/j.genhosppsych.2011.02.007 21601716

[4] Palacios J Khondoker M Mann A Tylee A Hotopf M. Depression and anxiety symptom trajectories in coronary heart disease: associations with measures of disability and impact on 3-year health care costs. J Psychosom Res. (2018) 104:1–8. 10.1016/j.jpsychores.2017.10.015 29275777

[5] Baumeister H Haschke A Munzinger M Hutter N Tully PJ. Inpatient and outpatient costs in patients with coronary artery disease and mental disorders: a systematic review. Biopsychosoc Med. (2015) 9:11. 10.1186/s13030-015-0039-z 25969694 PMC4427919

[6] National Audit of Cardiac Rehabilitation (NACR). Annual Report [Internet]. 2018 [cited 2023 July 12]. Available online at: https://www.bhf.org.uk/informationsupport/publications/statistics/national-audit-ofcardiac-rehabilitation-quality-and-outcomes-report-2018

[7] Bush DE Ziegelstein RC Tayback M Richter D Stevens S Zahalsky H Even minimal symptoms of depression increase mortality risk after acute myocardial infarction. Am J Cardiol. (2001) 88(4):337–41. 10.1016/s0002-9149(01)01675-7 11545750

[8] Hare DL Toukhsati SR Johansson P Jaarsma T. Depression and cardiovascular disease: a clinical review. Eur Heart J. (2014) 35(21):1365–72. 10.1093/eurheartj/eht462 24282187

[9] Rutledge T Linke SE Krantz DS Johnson BD Bittner V Eastwood JA Comorbid depression and anxiety symptoms as predictors of cardiovascular events: results from the NHLBI-sponsored women’s ischemia syndrome evaluation (WISE) study. Psychosom Med. (2009) 71(9):958–64. 10.1097/PSY.0b013e3181bd6062 19834049 PMC2783707

[10] Batelaan NM Seldenrijk A Bot M van Balkom AJ Penninx BW. Anxiety and new onset of cardiovascular disease: critical review and meta-analysis. Br J Psychiatry. (2016) 208(3):223–31. 10.1192/bjp.bp.114.156554 26932485

[11] Frasure-Smith N Lespérance F Talajic M. Depression and 18-month prognosis after myocardial infarction [published correction appears in circulation 1998 Feb 24;97(7):708]. Circulation. (1995) 91(4):999–1005. 10.1161/01.cir.91.4.999 7531624

[12] Barth J Schumacher M Herrmann-Lingen C. Depression as a risk factor for mortality in patients with coronary heart disease: a meta-analysis. Psychosom Med. (2004) 66(6):802–13. 10.1097/01.psy.0000146332.53619.b2 15564343

[13] van Dijk MR Utens EM Dulfer K Al-Qezweny MN van Geuns RJ Daemen J Depression and anxiety symptoms as predictors of mortality in PCI patients at 10 years of follow-up. Eur J Prev Cardiol. (2016) 23(5):552–8. 10.1177/2047487315571889 25665581

[14] Visseren FLJ Mach F Smulders YM Carballo D Koskinas KC Bäck M 2021 ESC guidelines on cardiovascular disease prevention in clinical practice. Eur Heart J. (2021) 42(34):3227–337. 10.1093/eurheartj/ehab484 34458905

[15] Sever S Harrison AS Doherty P. Levels of depressive symptoms in cardiac patients attending cardiac rehabilitation with a history of depression: pre COVID-19 and COVID-19 period comparison. BMC Cardiovasc Disord. (2022) 22(1):427. 10.1186/s12872-022-02867-4 36171545 PMC9517964

[16] Lespérance F Frasure-Smith N Koszycki D Laliberté MA van Zyl LT Baker B Effects of citalopram and interpersonal psychotherapy on depression in patients with coronary artery disease: the Canadian cardiac randomized evaluation of antidepressant and psychotherapy efficacy (CREATE) trial. JAMA. (2007) 297(4):367–79. 10.1001/jama.297.4.367 17244833

[17] Carney RM Blumenthal JA Freedland KE Youngblood M Veith RC Burg MM Depression and late mortality after myocardial infarction in the enhancing recovery in coronary heart disease (ENRICHD) study. Psychosom Med. (2004) 66(4):466–74. 10.1097/01.psy.0000133362.75075.a6 15272090

[18] Huffman JC Mastromauro CA Beach SR Celano CM DuBois CM Healy BC Collaborative care for depression and anxiety disorders in patients with recent cardiac events: the management of sadness and anxiety in cardiology (MOSAIC) randomized clinical trial. JAMA Intern Med. (2014) 174(6):927–35. 10.1001/jamainternmed.2014.739 24733277

[19] Davidson KW Bigger JT Burg MM Carney RM Chaplin WF Czajkowski S Centralized, stepped, patient preference-based treatment for patients with post-acute coronary syndrome depression: CODIACS vanguard randomized controlled trial. JAMA Intern Med. (2013) 173(11):997–1004. 10.1001/jamainternmed.2013.915 23471421 PMC3681929

[20] Richards SH Anderson L Jenkinson CE Whalley B Rees K Davies P Psychological interventions for coronary heart disease: Cochrane systematic review and meta-analysis. Eur J Cardiovasc Prev Cardio. (2018) 25(3):247–59. 10.1177/2047487317739978 29212370

[21] Blumenthal JA Sherwood A Smith PJ Watkins L Mabe S Kraus WE Enhancing cardiac rehabilitation with stress management training: a randomized, clinical efficacy trial. Circulation. (2016) 133(14):1341–50. 10.1161/CIRCULATIONAHA.115.018926 27045127 PMC4824555

[22] Wells A. Metacognitive Therapy for Anxiety and Depression. New York, NY: Guilford Press (2009).

[23] McPhillips R Salmon P Wells A Fisher P. Qualitative analysis of emotional distress in cardiac patients from the perspectives of cognitive behavioral and metacognitive theories: why might cognitive behavioral therapy have limited benefit, and might metacognitive therapy be more effective? Front Psychol. (2019) 9:2288. 10.3389/fpsyg.2018.02288 30662413 PMC6328488

[24] Wells A Reeves D Heal C Fisher P Davies L Heagerty A Establishing the feasibility of group metacognitive therapy for anxiety and depression in cardiac rehabilitation: a single-blind randomized pilot study. Front Psychiatry. (2020) 11:582. 10.3389/fpsyt.2020.00582 32714216 PMC7344162

[25] Wells A Reeves D Capobianco L Heal C Davies L Heagerty A Improving the effectiveness of psychological interventions for depression and anxiety in cardiac rehabilitation: PATHWAY-A single-blind, parallel, randomized, controlled trial of group metacognitive therapy. Circulation. (2021) 144(1):23–33. 10.1161/CIRCULATIONAHA.120.052428 34148379 PMC8247550

[26] Murray E Treweek S Pope C MacFarlane A Ballini L Dowrick C Normalisation process theory: a framework for developing, evaluating and implementing complex interventions. BMC Med. (2010) 8:63. 10.1186/1741-7015-8-63 20961442 PMC2978112

[27] May C. A rational model for assessing and evaluating complex interventions in health care. BMC Health Serv Res. (2006) 6:86. 10.1186/1472-6963-6-86 16827928 PMC1534030

[28] May CR Mair F Finch T MacFarlane A Dowrick C Treweek S Development of a theory of implementation and integration: normalization process theory. Implement Sci. (2009) 4:29. 10.1186/1748-5908-4-29 19460163 PMC2693517

[29] May C Finch T. Implementing, embedding, and integrating practices: an outline of normalization process theory. Sociology. (2009) 43:3. .org/10.1177/0038038509103208

[30] May C Finch T Mair F Ballini L Dowrick C Eccles M Understanding the implementation of complex interventions in health care: the normalization process model. BMC Health Serv Res. (2007) 7:148. 10.1186/1472-6963-7-148 17880693 PMC2089069

[31] May CR Mair FS Dowrick CF Finch TL. Process evaluation for complex interventions in primary care: understanding trials using the normalization process model. BMC Fam Pract. (2007) 8:42. 10.1186/1471-2296-8-42 17650326 PMC1950872

[32] Zigmond AS Snaith RP. The hospital anxiety and depression scale. Acta Psychiatr Scand. (1983) 67(6):361–70. 10.1111/j.1600-0447.1983.tb09716.x 6880820

[33] National Institute for Health and Care Excellence. Acute coronary syndromes [Internet]. 2020 [cited 2023 July 12]. Available online at: https://www.nice.org.uk/guidance/ng185

[34] National Institute for Health and Care Excellence. Chronic heart failure in adults: diagnosis and management [Internet]. 2018 [cited 2023 July 12]. Available online at: https://www.nice.org.uk/guidance/ng106 30645061

[35] Nelson EC Landgraf JM Hays RD Wasson JH Kirk JW. The functional status of patients. How can it be measured in physicians’ offices? Med Care. (1990) 28(12):1111–26. 10.1097/00005650-199012000-00001 2250496

[36] Wells A McNicol K Reeves D Salmon P Davies L Heagerty A Metacognitive therapy home-based self-help for cardiac rehabilitation patients experiencing anxiety and depressive symptoms: study protocol for a feasibility randomised controlled trial (PATHWAY Home-MCT). Trials. (2018) 19(1):444. 10.1186/s13063-018-2826-x 30115112 PMC6097432

[37] Braun V Clarke V. Using thematic analysis in psychology. Qual Res Psychol. (2006) 3(2):77–101. 10.1191/1478088706qp063oa

